# Online Occupational Therapy as a Rehabilitation Intervention for Parkinson’s Disease: A Systematized Review

**DOI:** 10.3390/clinpract15060098

**Published:** 2025-05-23

**Authors:** Antigoni Kountoura, Thomas Tegos, Marianthi Arnaoutoglou, Magdalini Tsolaki

**Affiliations:** 1 Department of Neurology, School of Medicine, Faculty of Health Sciences, Aristotle University of Thessaloniki, 54124 Thessaloniki, Greece; ttegos@auth.gr (T.T.); marnaout@auth.gr (M.A.); 2 Alzheimer Hellas, 54643 Thessaloniki, Greece; tsolakim@auth.gr

**Keywords:** Parkinson’s disease, online occupational therapy, telerehabilitation, quality of life

## Abstract

**Background/Objectives:** Occupational therapy (OT) plays a crucial role in addressing functional limitations and promoting independence in Parkinson’s disease (PD) patients. OT interventions target motor skills, daily activities, and engagement in meaningful tasks. Telehealth, the remote delivery of healthcare services, has expanded access to rehabilitation, including OT for PD. While several studies have examined the benefits of online OT, a comprehensive assessment of its impact on functional outcomes and quality of life (QoL) is needed. This review aimed to evaluate the effects of online OT interventions on functional outcomes and QoL of patients with PD. **Methods**: This review employed a systematized approach, guided by the Preferred Reporting Items for Systematic Reviews and Meta-Analyses (PRISMA) framework, though it did not constitute a full systematic review or meta-analysis. A comprehensive search was conducted across PubMed, Web of Science, Scopus, and Embase databases between August 2023 and September 2024. The search targeted randomized controlled trials (RCTs) investigating telerehabilitation interventions in OT for individuals with PD. Studies were excluded if they were not published in English, did not employ an RCT design, or lacked a focus on telerehabilitation within the scope of occupational therapy for PD. Additionally, systematic reviews, meta-analyses, qualitative studies, and studies without measurable outcomes were excluded. Nine studies met the inclusion criteria, with four involving occupational therapists directly and five evaluating interventions within the scope of OT practice. **Results**: The primary outcomes of this review focused on mobility improvements in PD patients, assessed through gait metrics such as gait speed, stride length, and gait variability. Secondary outcomes evaluated the impact of telerehabilitation on QoL, using tools such as the Parkinson’s Disease Questionnaire (PDQ-39) and other disease-specific instruments. The findings demonstrated that online OT interventions significantly improved motor skills, cognitive function, and activities of daily living in PD patients. Furthermore, these interventions enhanced overall well-being and QoL. The remote format fostered sustained engagement and adherence to therapy, contributing to better long-term outcomes. **Conclusions**: Online OT interventions show promising potential for improving functional outcomes and QoL in PD patients. These findings underscore the potential of telehealth to expand access to OT services, thereby enhancing long-term rehabilitation outcomes for this population.

## 1. Introduction

Recent advancements in electronic and telecommunications technologies have enabled occupational therapists to enhance patient care through telehealth. This umbrella term includes telemedicine and telerehabilitation, leveraging electronic communication for healthcare delivery. Telemedicine specifically focuses on improving patient outcomes through services such as assessment, prevention, diagnosis, and treatment through interactive technologies [1,2].

Within the scope of telehealth, therapists employ technology and electronic tools such as video conferencing to remotely conduct therapeutic sessions with clients. These services can take place either synchronously through online sessions using interactive technologies, or asynchronously through store-and-forward methods. Telehealth approaches also include assistive living technologies, encompassing tools and systems designed to enhance functional abilities for individuals with disabilities. These tools range from wheelchairs and chairlifts to adaptive aids like elastic bands that improve grip strength on items such as jars and mugs. Occupational therapists apply telehealth across various domains, including evaluations (tele-evaluation), interventions (tele-intervention), consultations (teleconsultation), client monitoring (tele-monitoring), and supervision (tele-supervision), using adaptive and rehabilitative technologies tailored for these purposes [2,3].

Telerehabilitation holds particular importance for individuals with Parkinson’s disease (PD), a condition characterized by progressive motor and non-motor symptoms that impair mobility, balance, and coordination. These challenges often make traveling to healthcare facilities difficult. Telerehabilitation addresses these barriers by delivering therapeutic interventions directly to patients’ homes, thereby improving access to essential care [4]. This approach aligns with the goals of occupational therapy, which seeks to enhance the quality of life for individuals with PD by addressing physical impairments and limitations in activities of daily living (ADLs) and instrumental activities of daily living (IADLs) [5].

For individuals with PD, quality of life is influenced by physical functioning, mental health, social participation, and the ability to perform daily activities—factors that significantly impact engagement in meaningful activities and social interactions [5,6,7,8]. As a chronic neurodegenerative disease, PD presents multifaceted challenges, including restricted mobility and difficulties leaving home, underscoring the need for innovative care solutions.

The growing global aging population has further amplified the demand for remote healthcare services, as many individuals with PD are older adults. Telerehabilitation offers flexible, adaptive care, enabling continuous monitoring and treatment without requiring in-person visits [9]. Additionally, the COVID-19 pandemic highlighted the importance of technology-based interventions as alternative care models, ensuring access to essential healthcare amidst disruptions to traditional service delivery [10]. Research indicates that utilizing telerehabilitation, compared to conventional intervention approaches, offers substantial advantages over conventional intervention approaches, including improved clinical outcomes, heightened engagement and completion rates, prolonged consultation durations, and increased client satisfaction levels [11]. With ongoing advancements in technology, integrating telehealth into occupational therapy has the potential to revolutionize service delivery and empower patients to achieve better health outcomes [12].

The integration of online occupational therapy interventions offers a promising solution for addressing the complex symptoms of PD while alleviating mobility-related challenges. These interventions can enhance quality of life and overall well-being, particularly when initiated early and sustained over time [13,14]. However, despite extensive research on telerehabilitation, challenges remain in developing comprehensive and standardized intervention programs due to methodological variations and limited evaluations of certain approaches [15,16]. Existing reviews have largely focused on services provided by healthcare professionals, including occupational therapists, but the findings on the impact of telerehabilitation on motor and non-motor symptoms, as well as quality of life, remain inconclusive [17,18,19,20].

Based on the above, this review aimed to identify and synthesize available research examining the effectiveness of telerehabilitation programs in individuals with PD. Specifically, it explored two primary research questions: (1) What technological tools and intervention methods are used in telerehabilitation for PD? (2) What are the benefits of telerehabilitation on the quality of life for individuals with PD?

By focusing on the specific role of occupational therapy in telerehabilitation, this review provides a unique perspective on PD management. It highlights how occupational therapists use telehealth platforms to deliver personalized, holistic care, improving patients’ quality of life and satisfaction. Additionally, the review emphasizes the need to standardize outcome measures and suggests future research directions, including integrating advanced technologies like virtual reality (VR) and artificial intelligence (AI). These insights pave the way for more comprehensive, accessible, and scalable telerehabilitation solutions for PD care.

## 2. Methods

### 2.1. Search Procedure

The PRISMA guidelines, accompanied by a valuable, simplified 24-step framework, were used in this review [21]. However, rather than conducting a full systematic review or meta-analysis, this study adopted a systematized approach. As a result, certain steps, such as PROSPERO protocol registration and standardized data extraction, were not completed prior to initiating the search. Instead, the primary goal of this review was to summarize and synthesize findings from relevant studies without statistically pooling data. In fact, the considerable heterogeneity in study designs, outcomes, and measurement tools across the included studies made statistical analysis impractical. To address this variability, a narrative synthesis approach was employed to qualitatively compare findings and identify emerging trends in the literature.

Given the absence of statistical synthesis, sensitivity analyses were not performed. Instead, the findings were assessed based on study quality and characteristics, highlighting observable patterns across studies with varying levels of methodological rigor. While formal statistical techniques were not applied, potential sources of heterogeneity—such as differences in intervention types, durations, and participant demographics—were explored narratively. For missing data, imputation was conducted only when reasonable assumptions, such as baseline similarities, could be applied to estimate outcomes. These considerations helped provide context for variations in study results without requiring quantitative analysis.

A comprehensive search was performed using PubMed, Scopus, Web of Science, and Embase databases. The search strategy targeted terms including the following: “occupational therapy”, “telerehabilitation”, “Parkinson’s disease”, “adult”, “quality of life”, and “QoL.” To ensure broad coverage, the search was later updated to include additional terms such as “virtual” and “augmented”, capturing a wider range of telemedicine studies, given the inherently virtual nature of telemedicine. Each term was expanded into related keywords, such as “telehealth” for telerehabilitation and “ergotherapy” for occupational therapy, to include all potentially relevant articles. A systematic search was conducted in each database, and additional search terms were tailored as needed for specific databases. All sources referenced in this review were last searched or consulted on 10 October 2024.

For example, the following terms were searched on Scopus: (TITLE-ABS-KEY (“occupational therap*” OR “ergotherapy*” OR “activity based therap*” OR “therapeutic occupation*” OR “Rehabilitation therap*” OR “occupational treatment” OR “rehabilitation treatment” OR “occupational rehabilitation” OR “therapeutic intervention” OR “occupation focused practice*” OR “OT” OR “occupation-al interventions” OR “rehabilitation interventions” OR “therapeutic activities” OR “rehabil-itation activities” OR “rehabilitation services” OR “rehab services” OR “telerehabilitation” OR “online rehabilitation” OR “remote rehabilitation” OR “e rehabilitation” OR “online occupational therapy” OR “remote occupational therapy” OR “teletherapy*” OR “remote therapy” OR “telehealth rehabilitation” OR “Virtual rehabilitation” OR “augmented reality interventions” OR “online therapy” OR “E-health rehabilitation” OR “virtual reality inter-ventio*” OR “VR Interventio*” OR “Virtual Reality Therapy” OR “VR Rehabilitation” OR “VR based Therapy” OR “VR assisted Rehabilitation” OR “Virtual Reality based Rehabili-tation” OR “VR based Interventions” OR “Digital Therapy” OR “Digital rehabilitation”) AND TITLE-ABS-KEY (“Parkinson’s disease” OR “PD” OR “Parkinson s” OR “Parkinson-ism” OR “Parkinson’s syndrome” OR “neurodegenerative disease” OR “Motor disorders” OR “Idiopathic Parkinson’s”) AND TITLE-ABS-KEY (“quality of life” OR “QoL” OR “well-being” OR “welfare” OR “functional independence” OR “Functional outcomes” OR “Health-related quality of life” OR “HRQoL” OR “Daily functioning” OR “Functional sta-tus” OR “Life satisfaction” OR “effectiveness” OR “efficiency” OR “efficacy” OR “benefit” OR “sufficiency” OR “competence” OR “influence” OR “value” OR “strength” OR “impact” OR “Influence” OR “Correlation” OR “Association” OR “Association” OR “Interaction”)) AND (LIMIT-TO (DOCTYPE, “ar”)) AND (LIMIT-TO (PUBYEAR, 2020) OR LIMIT-TO (PUBYEAR, 2021) OR LIMIT-TO (PUBYEAR, 2022) OR LIMIT-TO (PUBYEAR,2023) OR LIMIT-TO (PUBYEAR, 2024)) AND (LIMIT-TO (LANGUAGE, “English”)).

### 2.2. Eligibility Criteria

The eligibility criteria were established to include studies focusing on telehealth interventions in occupational therapy for patients with Parkinson’s disease. Only studies published within the last five years were included, reflecting recent advancements in telehealth technologies and the increasing adoption of telehealth for Parkinson’s rehabilitation. This timeframe also aligns with a pivotal period in telehealth adoption, driven by technological advancements and the broader availability of remote healthcare services.

Studies had to be published in English due to resource constraints for translation. Eligible studies focused on interventions specifically designed for individuals with Parkinson’s disease and delivered by qualified professionals. Randomized controlled trials, case reports, pilot studies, and other primary research designs reporting outcomes related to functional independence, symptom management, and quality of life were considered. Only articles fully accessible through online databases or inter-library loans were included in the review.

Among the eligible study designs, only RCTs were selected for this review as they provide the highest level of evidence for evaluating the effectiveness of interventions. RCTs offer controlled comparisons that are essential for establishing causal relationships between interventions and outcomes, directly addressing the research questions. Other primary study designs, such as observational or cohort studies, were excluded, as were meta-analyses and systematic reviews, since this review sought to analyze primary data rather than synthesize existing findings. Qualitative studies were also excluded, as the review focused on measurable outcomes such as motor function and QoL rather than patient experiences or perspectives. This approach allowed for a more precise assessment of the impact of specific telehealth interventions in occupational therapy for Parkinson’s disease.

Exclusions also extended to theses, case studies, editor notes, book reviews, letters, and pilot studies with missing data. Additionally, during the full-text review, articles were excluded if they did not pertain specifically to occupational therapy or interventions falling within its scope. Table 1 provides a detailed summary of the inclusion and exclusion criteria applied in the study selection process.

### 2.3. Review Parameters

Following the search, the study selection process was conducted as outlined below. All potential sources identified during the search were stored in a shared Zotero library, enabling all coauthors to access and review the articles. This centralized Zotero account ensured that no relevant studies were overlooked during the selection process.

The initial screening involved evaluating the titles and abstracts of the articles directly from the compiled dataset. Two coauthors independently reviewed the abstracts to determine whether each article met the inclusion criteria, were excluded, or required further review owing to insufficient information in the abstract. Articles flagged for further review were those in which eligibility could not be confidently assessed based on the abstracts alone.

Disagreements or uncertainties during this initial screening were resolved through group discussions among the coauthors. After the abstract screening, full-text articles flagged for further evaluation were thoroughly reviewed. The coauthors then collectively discussed and decided which articles met the predefined inclusion criteria and should be retained for the review, while excluding those that did not meet the requirements.

### 2.4. Data Collection

A structured approach was employed for data extraction, with a single reviewer systematically collecting relevant information from each selected article following a full-text review. Although standardized data extraction forms were not used, great care was taken to ensure accuracy and consistency across studies. Extracted data included participant characteristics, group assignment methods, study design, blinding procedures, intervention types, outcome assessments, compliance rates, alignment of interventions and controls, baseline similarity between groups, follow-up assessments, and the relationship between changes in patient well-being and the interventions.

**Figure 1:**
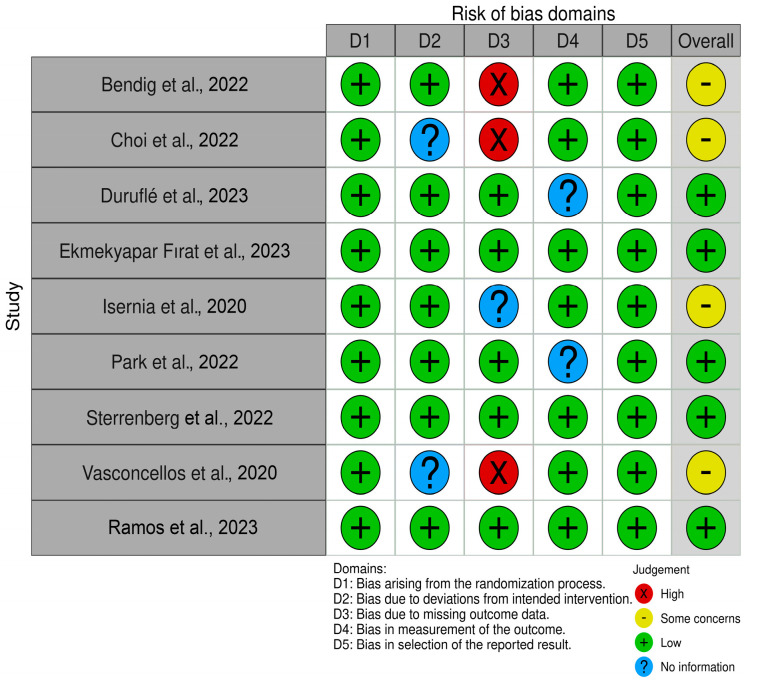
Risk of bias assessment of included studies based on Cochrane methodology, highlighting ratings across key domains (D1–D5) and overall judgment [5,9,22,23,24,25,26,27,28].

As all reviewed studies were published in English, translation was not required. Based on the risk of bias (ROB) assessment using the Cochrane methodology, five studies were rated as having an overall low risk of bias, reflecting strong methodological rigor. These studies adhered to best practices in areas such as the randomization process (D1), deviations from intended interventions (D2), and selection of the reported results (D5). In contrast, four studies were rated with “some concerns”, primarily because of missing data. While the overall quality of the included studies is robust, the identified concerns in a subset of studies warrant cautious interpretation of the findings. The results of the ROB assessment are summarized in Figure 1.

Additionally, certain limitations were noted, such as small sample sizes, which could impact the validity of some findings. Despite these limitations, the overall quality of the studies supports the conclusion that telehealth interventions in occupational therapy for Parkinson’s disease are promising and merit further investigation. To ensure a thorough evaluation of each study’s methodology and reporting quality, all bias assessments were conducted manually, as no automation tools were used.

For data synthesis, studies were grouped based on intervention types, outcome measures, and the technological tools utilized in telerehabilitation for PD. Where possible, studies with comparable control conditions—such as traditional in-person therapy or standard care without telerehabilitation—were grouped to facilitate comparisons of telerehabilitation’s effectiveness relative to conventional approaches.

Intervention characteristics were analyzed in detail, describing the components of each telemedicine program, including the integration of occupational therapy and PD-specific clinical management. The duration and modes of delivery, such as video conferencing, phone calls, mobile applications, and other digital technologies, were examined. Additionally, the qualifications and roles of healthcare providers were considered, alongside the specific aspects of PD management targeted by the interventions, such as gait training, medication management, and psychological support.

Study characteristics were further assessed by reviewing each study’s design to determine whether RCTs or other methodologies were employed. Funding sources were examined to identify potential biases or conflicts of interest, and the registration status of studies in clinical trial registries was verified to ensure adherence to research protocols and transparency.

For results analysis, the primary outcomes focused on mobility improvements, with particular attention to gait measures such as gait speed, stride length, and gait variability. Secondary outcomes included quality of life assessments, evaluated using disease-specific instruments like PDQ-39.

When information was missing or unclear, assumptions were made only when necessary and in alignment with common practices in the field (e.g., inferring an age range based on recruitment criteria when not explicitly reported). These assumptions were kept to a minimum to reduce potential bias in data interpretation and synthesis.

## 3. Results

A comprehensive search was conducted between August 2023 and September 2024, yielding a total of 7489 records from databases and hand-searching efforts. After removing 405 duplicate records and excluding 10 records deemed ineligible by automation tools, 7074 records remained for screening.

During the abstract and title screening process, 7050 records were excluded for not meeting the inclusion criteria. Articles were excluded at this stage if they lacked a randomized controlled trial design, focused on unrelated conditions or interventions, or did not provide outcome measures relevant to quality of life. The full-texts of the remaining 24 articles were reviewed, resulting in the exclusion of 14 additional articles that failed to meet the inclusion criteria.

**Figure 2:**
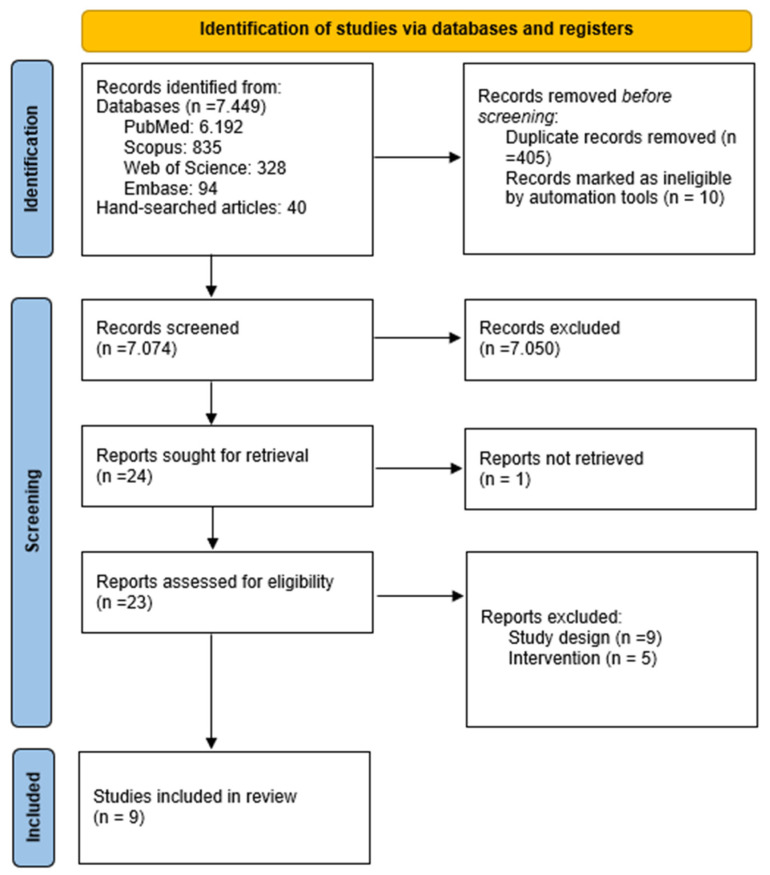
PRISMA flowchart of study selection process [29].

One report was not successfully retrieved due to accessibility issues, despite multiple retrieval attempts through inter-library loans and other resources. Consequently, nine articles were included in the systematic review, and relevant data were extracted and recorded. The detailed review process is illustrated in the flow diagram in Figure 2.

Most of the included studies clearly articulated their research questions and employed appropriate study designs. Methodologies were generally rigorous, with comprehensive descriptions of interventions and outcome measures. While certain limitations were noted, the overall quality of the reviewed studies supports the conclusion that telehealth interventions in occupational therapy for patients with Parkinson’s disease are promising and merit further investigation.

### 3.1. Participants

The participants in this review were individuals diagnosed with Parkinson’s disease across several included studies. Most studies focused on patients in the mild to moderate stages of the disease, classified as stages II-IV on the Hoehn and Yahr scale [5,22,23,24]. Participants were typically required to have a confirmed diagnosis of PD or clinically probable PD, as determined by a neurologist. In two studies, eligibility also included the ability to follow simple instructions, verified by a Mini-Mental Status Examination (MMSE) score of 20 or higher, to exclude individuals with significant cognitive impairment [23,24]. Furthermore, most studies excluded participants with comorbid neurological conditions other than PD or those with severe impairments that could hinder their participation in telerehabilitation interventions [5,22,23,24,25,26,27].

Recruitment methods varied, with participants often recruited from clinical settings [5] or rehabilitation centers [27]. Some studies also utilized remote recruitment strategies, such as advertisements through social media platforms [25]. The participant age range predominantly included middle-aged to older adults, as PD most commonly affects individuals aged 60 years and older.

Sample sizes across the studies ranged from small to moderate, with participant groups typically including between 12 [22] and 77 individuals [25]. Both intervention and control groups were incorporated where applicable, with control groups receiving either conventional rehabilitation [5,23,25,26,27] or no intervention [22,24] for comparison. The largest study included 77 participants recruited from general practitioners, nursing homes, or hospitals [25].

This careful participant selection enabled the review to examine the effects of occupational therapy telerehabilitation across a diverse spectrum of patients with PD. The focus remained on evaluating how these interventions could help maintain or improve quality of life through targeted therapeutic approaches.

### 3.2. Technological Equipment and Intervention Methods for Parkinson’s Disease in Telerehabilitation

The studies included in this review are summarized and presented in Table 2, which provides an overview of the key characteristics, methodologies, and findings of each study.

The technologies enabling telehealth services include synchronous videoconferencing, telephone communication, gaming devices for virtual reality activities, assistive devices, asynchronous text messaging, wearable sensors, mobile applications, and short video clips [5,22,24,25,26,27]. Among the nine studies, five utilized videoconferencing as a key telehealth communication method. This approach was employed to monitor patient progress, ensure safety during interventions, and provide instructions and follow-up care. The terms “telehealth” and “telerehabilitation” were often used interchangeably, referring to the practice of delivering rehabilitation through telehealth systems [5,22,23,24,25,27].

The integration of advanced technologies, such as mobile applications, smartwatches, and wearable devices, has shown considerable potential in enhancing patient engagement and outcomes. Programs like the HEAD Program and other comprehensive training initiatives have demonstrated significant improvements in both motor and cognitive functions through clinical and home-based interventions [23,27]. Remote monitoring tools, such as the TelePark App and wearable sensors, have further facilitated effective symptom management for Parkinson’s disease (PD) [22].

Specific interventions targeting trunk stability, physical performance, mobility, speech, and dexterity illustrate the potential of telehealth solutions in improving the quality of life for individuals with PD. App-based treatments, in particular, have consistently yielded positive outcomes in terms of motor and cognitive function and improved adherence to therapy in home-based settings [5,9,23,27]. Comparisons across studies reveal that videoconferencing remains one of the most widely used methods for direct interaction and support between therapists and patients [5,22,24].

The long-term benefits of teletherapy for Parkinson’s disease are particularly noteworthy, with studies suggesting sustained improvements in quality of life that extend well beyond the initial treatment phase [22,23,24,25,26,27,28]. Teletherapy not only provides temporary symptom relief but also establishes a foundation for consistent, patient-centered care. This approach supports functional independence, reduces the need for institutional care, and promotes long-term well-being [27]. However, the durability of these benefits depends on the ability of teletherapy to address both motor and non-motor symptoms as the disease progresses. Effective programs integrate structured follow-up protocols to sustain engagement and optimize therapeutic outcomes [5,26].

Furthermore, this review highlights significant improvements in patient satisfaction across various telehealth interventions for managing PD [5,9,22,23,24,25,26,27,28]. Satisfaction is closely linked to quality of life outcomes, especially in managing chronic conditions like Parkinson’s. Patients who are satisfied with their care often attribute this to the responsiveness and effectiveness of the interventions provided. In fact, personalized and comprehensive care strategies that address unique patient needs and preferences are strongly associated with higher satisfaction levels. For example, Duruflé et al. [25] demonstrated that enhanced patient support and coordinated care led to better health outcomes and emotional well-being. Similarly, Park et al. [24] found that the interactive features of mobile applications and smartwatches not only improved health management skills but also resulted in high satisfaction levels due to their engaging and supportive nature. These findings underscore the importance of tailored, multidisciplinary, and technology-enhanced approaches in achieving optimal quality of life for individuals with Parkinson’s.

Occupational therapists play a pivotal role in the success of telehealth interventions for PD. They coordinate multidisciplinary approaches, validate the use of technical aids, and provide continuous support to both patients and caregivers [24,25,28]. The reviewed studies further emphasize the effectiveness of telehealth and telerehabilitation in managing chronic conditions, particularly in rural and underserved areas. The diverse methodologies and technologies employed demonstrate the versatility of these interventions.

Despite these promising findings, the consistent positive outcomes—such as improved health measures, patient engagement, and satisfaction—highlight the need for continued research and development in this field. Expanding sample sizes in future studies and further exploring the integration of innovative technologies will be essential for advancing telehealth’s potential in managing Parkinson’s disease effectively.

### 3.3. Benefits of Telerehabilitation on Quality of Life in Parkinson’s Disease Patients

The included studies evaluated the effectiveness of telehealth interventions on the quality of life of patients with Parkinson’s disease. Overall, the majority of participants reported positive experiences, with improvements reflected in QoL feedback across all studies [5,22,23,24,25,27].

One of the key advantages of telehealth is its ability to provide consistent therapy without the geographical or logistical barriers often faced by PD patients. This accessibility allows individuals to benefit from interventions that address both motor and non-motor symptoms, ultimately enhancing their overall well-being. For instance, telerehabilitation programs such as LSVT-BIG [26] and the HEAD Program [23,27] have demonstrated significant improvements in motor function, activities of daily living, and non-motor experiences, including mood and cognition.

Several studies reported a positive correlation between motor function improvements and QoL scores following telehealth interventions. Patients participating in telerehabilitation exercises showed notable advancements in mobility [5,22,23,24,25,26,27,28], balance [5,23,24,27,28], upper limb function [27], and psychological well-being [22,23,25,26]. Interventions also reduced symptoms of anxiety and depression, contributing to better overall mental health [24,25,27,28].

The findings of Ramos et al. [9] reinforce these benefits, particularly in underrepresented community settings. Their study demonstrated that telerehabilitation effectively reduced mobility limitations, as evidenced by improved Time Up and Go test results, and enhanced QoL even in low-resource environments. This highlights the versatility and adaptability of telehealth interventions across diverse populations.

However, certain limitations were observed. For instance, trunk-specific exercises alone did not consistently outperform traditional limb-focused exercises [5,22], suggesting that a comprehensive approach combining multiple types of exercises might be necessary to optimize outcomes. Psychological improvements, particularly reductions in anxiety and depression, were most pronounced in interventions utilizing mobile health tools that enabled continuous communication and feedback [24,25]. These findings emphasize the importance of addressing both motor and non-motor symptoms in telehealth care strategies.

The integration of virtual reality in some programs has also enhanced patient engagement and motivation, further improving adherence to exercise regimens [27]. Adherence is critical, as studies have consistently shown a direct relationship between regular participation in telerehabilitation and improvements in both mental and physical health.

From an economic perspective, telehealth interventions offer significant potential to reduce immediate costs and achieve long-term savings. Benefits include enhanced symptom management, reduced caregiver strain, fewer hospitalizations, improved care accessibility, and alleviated burdens on families and the healthcare system [22,23,25,26,27]. However, adherence remains a key factor in realizing these economic advantages [5].

Studies also demonstrated favorable cost-effectiveness ratios for teletherapy. For instance, Duruflé et al. [25] reported a positive incremental cost-effectiveness ratio for telerehabilitation, indicating its financial advantage compared to traditional face-to-face care. This was especially evident in milder cases, where equivalent therapeutic outcomes were achieved at reduced costs. Similarly, Isernia et al. [27] showed that home-based telerehabilitation could prevent costly hospitalizations and institutional care by enabling patients to maintain autonomy and well-being in their homes.

Overall, these improvements contributed to better QoL and reduced social restrictions associated with PD. The findings underscore the importance of multidisciplinary, comprehensive care strategies that address both motor and non-motor symptoms [30]. To further optimize long-term outcomes and economic impacts, adherence challenges must be addressed through improved patient engagement and regular follow-up protocols. Enhancing adherence could strengthen the cost-effectiveness of teletherapy, solidifying its role as a sustainable and impactful approach to managing Parkinson’s disease.

## 4. Discussion

The findings of this review emphasize the value of occupational therapy telerehabilitation in enhancing the quality of life for individuals with Parkinson’s disease. The included studies illustrate a variety of telerehabilitation approaches, demonstrating how telehealth platforms effectively address both motor and non-motor symptoms of PD. Common outcomes across the nine studies included high patient satisfaction and improved QoL, achieved through tailored therapeutic interventions that focused on motor control, cognitive function, and mood [5,22,23,25].

A key advantage of telerehabilitation is its ability to deliver consistent, remote care, overcoming the geographical and mobility constraints that often hinder access to traditional rehabilitation services. Studies have shown that telerehabilitation can significantly improve motor functions such as balance and mobility, while also positively affecting non-motor symptoms like mood, cognition, and psychological health [5,23,27]. For example, programs such as LSVT-BIG and the HEAD Program achieved substantial gains in motor control, which directly correlated with QoL improvements [26,27].

Telerehabilitation’s economic impact is another notable finding. By reducing the need for frequent in-person interventions, it minimizes healthcare expenses, including costs associated with hospital visits, transportation, and reliance on physical healthcare infrastructure. These cost savings are especially beneficial in underserved regions where access to specialized care is limited [9,25,27]. The scalability of telerehabilitation further supports its inclusion in healthcare policies and reimbursement models, as it is both cost-effective and capable of improving patient outcomes.

Patient satisfaction emerged as a recurring theme, closely linked to the customization and flexibility inherent in telerehabilitation interventions. High satisfaction levels were attributed to the interactive nature of telehealth platforms, which facilitate personalized care through mobile applications and wearable devices that enable real-time monitoring and feedback [22,24,25]. For instance, Park et al. [24] observed that the interactive design of mobile technologies enhanced patient engagement and self-management, contributing to greater satisfaction. The ability to tailor telerehabilitation to individual needs and preferences was a key factor in achieving these positive outcomes.

This review also highlights the importance of continuity and structured follow-up in realizing the long-term benefits of telerehabilitation. Effective programs incorporated follow-up protocols to maintain patient engagement and sustain therapeutic outcomes, fostering functional independence and reducing reliance on institutional support over time [5,26]. Aligning patient satisfaction with sustained adaptive care is critical to addressing the evolving needs of individuals with PD.

Occupational therapists play a pivotal role in PD telerehabilitation, coordinating multidisciplinary approaches and providing essential support to patients and caregivers. These professionals guide patients through personalized interventions, validate the use of technical aids, and ensure the effective integration of technology with clinical expertise [24,25,28]. A multidisciplinary approach, combining therapeutic insights with technological tools, is essential to maximize the benefits of telerehabilitation for PD patients.

This review highlights promising findings for telerehabilitation in Parkinson’s disease but also underscores several limitations. Small sample sizes, with only one study exceeding 77 participants, limit generalizability, while the absence of long-term follow-up data hinders evaluation of sustained benefits. Variability in PD severity among participants and inconsistencies in outcome measures further complicates direct comparisons. Additionally, reliance on English-language studies may introduce language bias, excluding relevant findings from non-English sources.

Participant demographics showed wide variability in female representation (35.7% to 69.8%), emphasizing the need for balanced gender representation in future research. Although the included studies demonstrated moderate-to-high methodological rigor, some were affected by missing data and potential biases, warranting cautious interpretation of the findings.

Future research should address these limitations by employing standardized outcome measures, larger and more representative sample sizes, and multilingual studies to improve their applicability. Bridging barriers to adoption, such as limited access to technology, low digital literacy, and socioeconomic disparities, is critical. Additionally, training healthcare providers and integrating advanced tools like virtual reality, wearable devices, and artificial intelligence can further enhance telerehabilitation’s effectiveness and accessibility.

By addressing these challenges, telerehabilitation has the potential to transform PD management through its adaptability, cost-effectiveness, and ability to reach diverse populations, ultimately improving the quality of life of PD patients.

These findings have significant implications for clinical practice, policy, and research. Clinicians might consider integrating telerehabilitation as a complement to in-person therapy, especially for patients with limited mobility or those in rural areas. Multimodal telerehabilitation interventions that address both motor and cognitive functions can maximize treatment benefits. For policymakers, investments in digital health infrastructure and the development of supportive reimbursement policies could make telerehabilitation more accessible and affordable for PD patients [23]. Research should continue to standardize outcome measures, conduct large-scale studies, and explore advanced technologies like artificial intelligence and virtual reality to enhance the effectiveness of telerehabilitation.

In conclusion, this review demonstrates that occupational therapy telerehabilitation provides significant benefits for individuals with PD by improving QoL and complementing traditional therapies. By enabling patients to reinforce their skills remotely, telerehabilitation supports continuous care, promotes functional independence, and optimizes therapeutic outcomes through a hybrid model of in-person and remote sessions. Its adaptability and structured follow-up protocols make telerehabilitation a promising tool for PD management, with the potential to advance clinical practice and improve the overall well-being of patients with Parkinson’s disease.

## Figures and Tables

**Table 1:** Inclusion and exclusion criteria.

Elements	Inclusion	Exclusion
Population	Patients diagnosed with Parkinson’s disease, primarily in mild to moderate stages (Hoehn and Yahr scale II–IV).	Studies with participants aged <19 People with functional limitations
Interventions	Telerehabilitation programs for occupational therapy, including technologies like video conferencing, mobile apps, wearable sensors, virtual reality, and specific therapeutic exercises.	Art therapy Music therapy Dance/movement therapy Animal-assisted therapy Horticulture therapy Recreational therapy Play therapy Aquatic therapy
Comparison	Traditional in-person therapy or no intervention in control groups across the studies.	
Outcome	Improvements in motor functions (e.g., gait speed, balance, mobility), non-motor symptoms (e.g., anxiety, depression, cognition), quality of life, adherence to therapy, and patient satisfaction.	
Study design	Randomized control trials, pilot studies with results.	Theses and/or case studies Systematic reviews and meta-analyses Editor’s note Book reviews and/or letters Qualitative studies Pilot studies with no results
Date restrictions	The search was conducted for studies published between 1 January 2019 and 10 October 2024.	
Language restrictions	English language.	Other languages

**Table 2:** The features of the studies included in this review.

Authors	Sample	Intervention	Outcome	Conclusion
Bendig et al. [22]	11 participants (45% female, 55% male); clinically probable diagnosis of PD	TelePark App and Patient Management Platform; video visits; wearable sensors (PD Monitor); camera system (Motognosis Amsa Software)	Quality of life, medication support, participant adherence	Telehealth interventions improved QoL and provided effective symptom monitoring but did not significantly enhance mobility outcomes
Choi et al. [23]	60 participants with PD diagnosis (gender distribution information for participants is not provided); 30 participants in the control group and 30 participants in the intervention group	Multimodal Rehabilitation Intervention: Daily life training, home environment modification, fine muscle exercises, fall prevention exercises, Guardian education; traditional rehabilitation: task-oriented training, joint exercise, ADL training follow-up	Daily life behavior; QoL in PD patients; burden of care felt by guardians of PD patients	Multimodal telerehabilitation improved QoL and daily functioning, benefiting both patients and caregivers
Duruflé et al. [25]	77 participants (gender distribution data shows a discrepancy, preventing accurate percentage calculations); patients with severe neurological disabilities (including Parkinson’s disease); 38 participants in the control group (EMR2) and 39 in the experimental group (TELEMED)	EMR2 (Mobile Rehabilitation Team): Assess patients and their environment, establish care objectives, coordinate with medical contacts, provide support; TELEMED (experimental group): identical medical intervention and assessment as control group with teleconsultation follow-up	Medical–Economic Analysis; level of goal attainment; anxiety and depression; pain; caregiver burden; participant satisfaction	Telerehabilitation reduced anxiety, depression, and caregiver burden, leading to high patient satisfaction
Ekmekyapar Fırat et al. [26]	15 PD Patients (40% female, 60% male) diagnosed according to UK Parkinson’s Disease Association Brain Bank Clinical Diagnostic Criteria	Lee Silverman Voice Therapy (LSVT)-BIG: LSVT-BIG telerehabilitation program, consisting of 16 remote sessions over 4 weeks, was used to assess its impact on quality of life. Motor and non-motor experiences were evaluated before and after using MDS-UPDRS parts 1, 2, and 3, along with quality of life measured by PDQ-39	Significant improvements were noted in motor symptoms (such as gait and balance) and non-motor symptoms (such as emotional well-being), as well as in overall quality of life, except for the social support section of PDQ-39	LSVT-BIG telerehabilitation significantly improved motor function and QoL, especially in motor symptoms
Isernia et al. [27]	31 people (45.2% female, 54.8% male) with PD (1 month of Clinic HEAD rehabilitation); 11 patients allocated to HEAD rehabilitation; 20 people with PD were included in the UC condition (3 patients in UC group were not evaluated)	HEAD Program: Multidimensional rehabilitation for enhancing motor and cognitive functions in chronic neurological diseases (1 month Clinic HEAD + 3 months Home HEAD); Usual Care (UC) Condition: no additional motor or cognitive rehabilitation activities, health recommendations from neurologists	Motor and non-motor symptoms; quality of life	The HEAD Program improved both motor and cognitive functions, leading to enhanced QoL
Park et al. [24]	43 participants (69.8% female, 30.2% male); people with PD diagnosis; 23 participants in the control group and 20 participants in the intervention group	Mobile Health Intervention based on IMB Model: Mobile applications, smartwatches, smartphone-based short text messages, information messages, telephone counseling; control group: short text messages, telephone counseling	Motor Functions Assessment: Static balance, risk of falling, walking speed, distance walked, upper extremity function; non-motor functions assessment: Executive function, memory, language, visual–spatial abilities; QoL and psychological well-being assessment; positive affect and negative affect states	The mobile health intervention improved psychological well-being and mobility, enhancing overall QoL
Sterrenberg [28]	4 participants, including 2 males (50%) and 2 females (50%), completed the full 10-week program	A 10-week education and wellness program was delivered to five participants by a multidisciplinary team, led by an occupational therapist. The program included 1 education session and 5 wellness sessions per week, totaling 60 sessions. Each session lasted one hour. Education sessions, led by the occupational therapist, covered daily living strategies and future planning, while wellness sessions, conducted by various allied health professionals, included cognitive and physical challenges	Self-efficacy; motor symptoms; non-motor symptoms; self-management of PD; QoL	Multidisciplinary wellness programs improved self-efficacy, motor symptoms, and QoL
Vasconsellos et al. [5]	28 individuals with PD (35.7% female, 64.3% male); 14 in the control group and 14 in the experimental group	Control Group Intervention: Upper and lower limb exercises, home-based; experimental group intervention: trunk exercise program, home-based	Primary outcomes are focused on improvements in QoL for individuals with Parkinson’s disease	Trunk exercises improved QoL but were less effective than comprehensive motor exercises for improving gait and balance
Ramos et al. [9]	19 participants (47.4% female, 52.6% male) (Brazilian Amazon)	Telerehabilitation program vs. booklet-based exercise program for 4 weeks	Adherence, safety (no major adverse effects)	Telerehabilitation improved mobility and QoL, with high adherence and minimal adverse effects, even in low-resource settings

## Data Availability

The data supporting the findings of this study are available from the corresponding author upon reasonable request.
